# Understanding chemical reactions through multimedia resolution data artistic educational tool ( *MRDAET* )

**DOI:** 10.1002/pro.70648

**Published:** 2026-06-02

**Authors:** Hana Pokojná, Barbora Kozlíková, Katarína Furmanová, Adam Štěpánek, Simone Kriglstein

**Affiliations:** ^1^ Faculty of Informatics Masaryk University Brno Czech Republic; ^2^ Austrian Institute of Technology GmbHs Vienna Austria

**Keywords:** biochemistry education, biomedical art, interactive education, multimedia installation, public outreach, science communication, scientific visualization

## Abstract

Through the integration of science, art, and technology, we present Multimedia Resolution Data Artistic Educational Tool (*MRDAET*), an interactive installation designed to support the visualization and understanding of molecular reactions. The installation focuses on ATP synthesis and the electron transport chain—core biochemical processes that are often difficult to grasp due to their complexity and abstract nature. Inspired by David Goodsell's fusion of scientific accuracy and artistic expression, *MRDAET* employs physical 3D models derived from structural data in the Protein Data Bank, augmented with projection mapping and enhanced with illustrations. The installation encourages users to explore molecular processes through layered interactivity that combines tangible models with animated visual overlays. *MRDAET* was evaluated at an art and technology event, where user feedback indicated that the installation was both enjoyable and perceived as helpful in education. Participants reported improved understanding of the presented biochemical concepts and expressed interest in increased interactivity, particularly through Mixed Reality integration connecting physical models and dynamic animations. While individual and combined visual modalities have been shown to be effective in science education, immersive interactive installations of this type remain underexplored in this context. This paper presents *MRDAET* as the third iteration following prior designs, offering a science‐driven educational approach that warrants further study and application to additional scientific areas.

## 1 INTRODUCTION

Biochemical education presents specific challenges, including the need to reason about microscopic particles that are invisible to the naked eye and complex processes that often occur simultaneously. Biochemistry classes are a mandatory part of European curricula (Jones et al., 2014); however, high school students often experience difficulties in understanding this discipline (Dreyfus et al., 2014; Gayford, 1986). This issues arise from limited understanding of spatial arrangements (Wu & Shah, 2004), the integration of biology and chemistry, and the inherently abstract nature of the subject (Green et al., 2021). To address this, we developed a multimodal tool aimed at enhancing comprehension of biochemistry, designed not only for high school students but for a broader audience as well.

The issues with understanding these complex topics were successfully approached by a biochemist and molecular artist, David Goodsell, whose watercolor paintings revolutionized visualization of crowded cellular spaces. By marrying art with science, Goodsell's visualizations distinguish components within the cell and foreshadow their function through color (Goodsell, 2016). The science‐based product also serves as an aesthetic piece that mirrors real life through artistic expression. Earlier examples of Goodsell's work (Goodsell, 2009) showcase the perfect balance between science and art that benefits both disciplines. His approach to using color that harmonizes with function or hierarchical division of molecules within a cell brought to life so‐called “Goodsell coloring,” which is nowadays used in applications for biochemical visualization and education, for example, by Johnson et al. (2015) and Alharbi et al. (2023).

Previous empirical evidence has shown that concrete examples of chemical compounds, such as physical models (e.g., double‐helix (Watson & Crick, 1953)) and computer‐generated visualizations (Rayan & Rayan, 2017), improve spatial understanding of biochemical data (Ferk et al., 2003). Accessibility to understanding abstract scientific concepts can be achieved through art, as demonstrated by Goodsell's work, (e.g., Goodsell, 2009; Goodsell, 2016). Visual media have been proven to be helpful in facilitating both education and outreach, especially because molecular occurrences that happen on a smaller level can be difficult to grasp as people do not always understand the relationship between the cells, molecules, atoms, and the timescales at which these structures are working in Jenkinson (2018). To improve understanding of scientific concepts, scientific literacy needs to be improved, for example through combining multiple modalities and supporting students' engagement with topics (Krajcik & Sutherland, 2010).

In this study, we depict the adenosine triphosphate (ATP) synthesis and its closely related preceding process, the electron transport chain, a series of molecules that carry out chemical reactions providing molecules for ATP Synthesis. In our prototype installation, called Multimedia Resolution Data Artistic Educational Tool—*MRDAET* for short—we designed a mixture of 2D illustrations, 3D interactive movable physicalizations, and 2.5D animation that overlays 3D physical models to recreate the human cell. While the first two iterations (Pokojná, Kriglstein, et al., 2024a) of this project, briefly described in Section 3, focused on mechanism of the main model (ATP synthase) and the interactions of the surrounding molecules (electron transport chain), this iteration aims to help with understanding the electron exchange, molecular grouping by function, as well as spatial placement of the organelles and molecules within the cell. This is achieved by layering interactivity with the visualization and combining multiple modalities. In this design, the learner “enters” the cell at the lowest magnification and continues to “zoom in” to the mitochondrial wall, where the electron transport chain and ATP synthase are located. With increased magnification, the design increases in interaction, that is, starting with 2D illustrations and ending with interaction with physicalizations—3D physical and tangible representations of data (Jansen et al., 2015). Each of the iterations increases with the complexity (Norooz et al., 2015) of the chemical concepts and visualizations that explain them. This combination was decided based on conducted user studies and previous research into illustrations, animation, and physicalizations, and aims to harvest the benefits of each modality. This paper describes the third iteration and prototype of immersive experience with layered interactivity and evaluates the following research questions (RQs):

*In what ways does multi‐modal, data‐based art installation with different levels of interaction influence users' enjoyment when learning about complex chemical reactions?*

*In what ways is a multi‐modal, data‐based art installation with different levels of interaction perceived as helpful in learning about complex chemical reactions?*

We have investigated these questions by exhibiting *MRDAET* at a popular science, art, and technology event, a setting where science creates art and art is based on science, similarly to Goodsell's molecular art ethos. With our subsequent survey‐based study, we demonstrate the applicability and feasibility of installation, such as *MRDAET*, and that it provides an alternative direction to presenting scientific concepts. Because our study focused on testing the prototype and whether this type of visualization is worthy of investigating further, we offer recommendations for future and more controlled research on visualizations similar to *MRDAET*, informed by our observations.

## 2 RELATED WORK

This section discusses the research related to the importance of Science, Technology, Engineering, Arts, and Mathematics (STEAM) education that extends beyond classrooms. Learning can take place in museums or science festivals and focuses on the subject by leveraging the power of visualization and human–computer interactions (HCI). We then follow with insights into the benefits of each modality and the benefits of using art to enhance science.

Museums are becoming places to interact with science rather than passively consume information (Sayffaerth et al., 2024). They provide a new type of education through arts and interaction, successfully teaching science subjects due to the interdisciplinary approach to communicating information (Lin et al., 2019). A great example is the field of medical art, which facilitates the understanding of complex biomedical subjects through the variety of modalities it provides (Erolin, 2020), such as 3D digital models, 3D physical models, animations, and illustrations. The principles of STEAM education also relate to the notion of “Exploranation,” an approach tailored for museums where knowledge is encouraged to be learned through exploration of real data, which can be presented through the use of standard GPU renderings (Ynnerman et al., 2018). Another commonly employed approach in playful education is the concept of “embodied experience,” where learning is enhanced by physically interacting with abstract concepts, which allows learners to connect mental representations with tangible actions (Reiner, 1999). In this paper, we adhere to all three, STEAM, Exploranation, and embodied experience principles, by using real data, making them visually appealing *and* tangible, while not utilizing immense computational power. An example of an immersive educational institution that heavily relies on art and technology to help understand science through visualizations is museum ARTIS‐Micropia (ARTIS Zoo, 2014) in Amsterdam (Netherlands). This museum provides understanding of the invisible world of microbes around us through various types of visualizations. These range from interactive applications, through the use of microscopes, projections, 3D physical models, animated projections, and even artistic pieces like *The Fungal Wall* (2021) by Lizan Freijsen, showing mold‐inspired hand‐tufted large‐scale installation on the wall, or the *Glass Microbiology* by Luke Jerram, the glass‐blown transparent sculptures of viruses such as Ebola.

**FIGURE 1:**
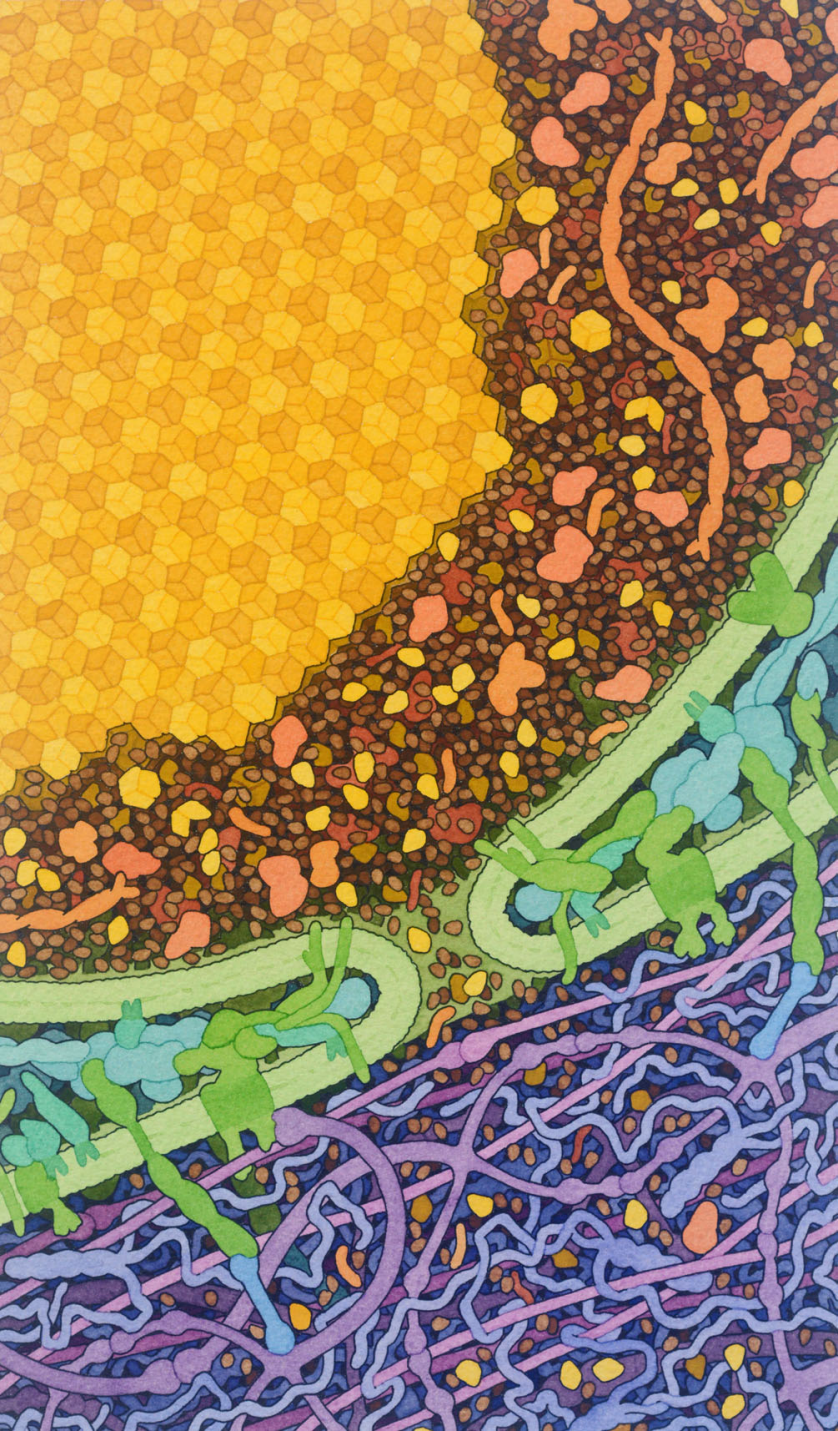
Goodsell's illustration of Insulin Release (2022), demonstrating insulin crystals being carried to the extracellular space in vesicles. This image (Goodsell, 2022) is one of many in the Molecular Landscapes series, published on the RCSB Protein Data Bank and Scripps Research. Image used for educational purposes under CC‐BY‐4.0 license.

Museum‐based visualizations present science through approaches that contrast with the conventional instruction commonly used in schools. The standard 2‐dimensional (2D) written formulas are often used primarily in educational setting to represent chemical compounds and chemical reactions, which differ from the enhanced 2D renders, for example, in Goodsell's detailed illustrations. As an example, consider insulin export from the cell. Standard written formula for describing the structure of insulin monomer alone ($C_{257}H_{383}N_{65}O_{77}S_{6}$) may come across a lot less visually appealing than a picture showing the same molecule. Both the written formula and the accompanying image provide a visual description of molecular structures and their interactions. Yet looking at the image (Figure 1) showing insulin being processed in a cell, one can clearly tell the various structures apart by color grouping and spatial placement within the cell (e.g., intra‐ and extracellular matrices) due to composition. Despite the illustration being a static image, the movement of vesicles coming towards the cell membrane and exiting the cell is clearly shown. While the standard written formulas should not be replaced by artistic renders alone, individuals without a background in chemistry may be cognitively overwhelmed and potentially discouraged to learn about the topic when presented with the formulas alone. On the other hand, being presented with an image of crystals of insulin carried in vesicles from pancreatic beta cells to the extracellular matrix may enhance understanding of the concept *and* spark interest in the topic due to its aesthetic depiction of real data, which would lead to studying more standardized ways of noting them.

Illustrations have been the original way of representing scientific concepts, for example, Johannes Kepler's sphere arrangements (Kepler, 1966) in six‐sided starlets making up a snowflake, which was a predecessor to molecular illustration. Molecular and cellular illustrations, other than the traditional watercolors by David Goodsell, can also be rendered digitally based on real data. With 3D‐modeling and animation software and plugins, such as Molecular Maya (mMaya) (mMaya, n.d.) or BioBlender (BioBlender, 2025) specifically made for accurate visual representation, molecular visualizations can be made with cinematic quality.

Entire cellular landscapes (e.g., when utilizing cellPack (Johnson et al., 2015)) or standalone molecules can be visualized based on their raw Protein Data Bank (PDB) data (Berman et al., 2000), obtained from a global database of experimental structures. Their structural architecture and movements can be presented through 3D digital models in animations (Jenkinson & McGill, 2012; Iwasa, 2015). Examples include the works of Drew Berry (Berry, 2007; Berry, 2015; Berry & Tétaz, 2018), that, similarly to Goodsell's work, leverage art to popularize science and make art educational. Numerous studies have shown that molecular animations help students grasp bimolecular concepts that are otherwise unseen to the naked eye (Jenkinson, 2018; McClean et al., 2005). These visual aids can take many forms, from videos to illustrations segmented to various lengths and levels of detail to fit their target audience (Pokojná, Kozlíková, et al., 2023a). These can even be explained through a visual metaphor, such as showing metabolic pathways as a city, making the science more understandable and enjoyable (Adnan, 2020), a strategy that is often used in the designing of scientific infographics (Pokojná et al., 2025). While infographics are often used to make science more available to novices, the use of animation can also simulate data to help visualize hypotheses (Nayak et al., 2020).

Other educational modalities include 3‐dimensional (3D) physical models, such as sticks‐and‐balls models, that are standardly used in high schools to visualize molecular structures (Copolo & Hounshell, 1995). A more modern way of creating physicalizations is to 3D print (Gardner & Olson, 2016) the scientifically accurate structures also obtained from the PDB data (Berman et al., 2000). Scientific 3D prints already gained popularity in education (Minshall & Ford, 2019). Accurate molecular 3D physical models can be designed to move and represent molecular movements by adjusting them in 3D modeling software, for example, Blender (Online Community Blender, 2018), to represent spinning of the motor (Pokojná, Kriglstein, et al., 2024a) or docking (Alderighi et al., 2021). Similarly to the concept of making science accessible through 2D art as in Goodsell's paintings, data sculptures provide information through 3D art by bridging different areas of knowledge, for example, information visualization and art, among others (Dragicevic et al., 2021). The use of physicalizations is especially useful as it facilitates learning through making abstract concepts visible (Norooz et al., 2015). This is also relevant to the embodied learning (Reiner, 1999), a different teaching method that uses the same principle—making abstract concepts more concrete—through guided and purposeful body movements (Abrahamson & Lindgren, 2014). Physicalizations can also be leveraged by being situated within the target context (Perovich et al., 2021), a project where data were visualized and made more accessible through physicalization, which was placed within the environmental context (polluted creek), making it a site‐specific event. Their other benefit is to provide a collaborative learning environment (Shaer & Hornecker, 2010), which contributes to social, psychological, academic, and assessment‐related benefits (Laal & Ghodsi, 2012). Their interactive properties also aid in understanding dynamic processes that change over time (Pahr et al., 2024). The multi‐sensory input helps with spatial reasoning and later recall (Jansen et al., 2015), which ultimately supports learning.

Technology today also provides digital enhancements that help us understand these complex biochemical structures through Augmented Reality (AR) (Woźniak et al., 2020; Irwansyah et al., 2018) and Virtual Reality (VR) (Kut'ák et al., 2021; Ramírez & Bueno, 2020). The design of the virtual environments goes beyond individual experiences by presenting collaborative virtual environments allowing exploration of molecular worlds (Kut'ák et al., 2021; Pokojná, Rasheed, & Schönborn, 2023b).

The above outlined benefits of individual modalities (illustrations, animation, physicalizations, and even mixed reality (MR)) make a significant contribution to learning, particularly in STEM disciplines, where they facilitate the understanding of abstract concepts. While the modalities have been studied individually or compared with each other, to our knowledge, their combination in the context of biochemistry has not been researched yet. Investigations of individual modalities also suggests (Krajcik & Sutherland, 2010; Pokojná, Kozlíková, et al., 2023a) that layering multiple modalities can be beneficial to learners. Therefore, we designed *MRDAET* to examine how participants respond to such an art installation. The research methods used to evaluate this prototype were similar to other studies investigating novel visualizations in biomedicine for public outreach (Pokojná, Kriglstein, et al., 2024a; Pokojná, Kozlíková, et al., 2024b) and are further discussed in Section 4.2.

## 3 DESIGN OF *MRDAET*

In the following section, we explain why we selected these specific chemical reactions, describe the previous two design iterations presented in our earlier publication (Pokojná, Kriglstein, et al., 2024a), and justify the design decisions of the current iteration.

### 3.1 Why ATP production

The mitochondria, colloquially referred to as the “Powerhouse of the cell,” became the focus for *MRDAET* visualization as it is the site of ATP production. Because of its essential part in the high school curriculum, we assumed its importance as general knowledge. We previously investigated the best practices to represent chemical reactions (and used the ATP synthesis as a test subject) in 2D in the form of illustrations and animations (Pokojná, Kozlíková, et al., 2023a). We then tested two iterations of 3D physicalizations (Pokojná, Kriglstein, et al., 2024a) that yielded this third iteration. This study continues to explore the best way to present complex information by combining various modalities and their benefits to make information retention more accessible for novices in biochemistry.

### 3.2 Biochemistry illustrated in *MRDAET*

**FIGURE 2:**
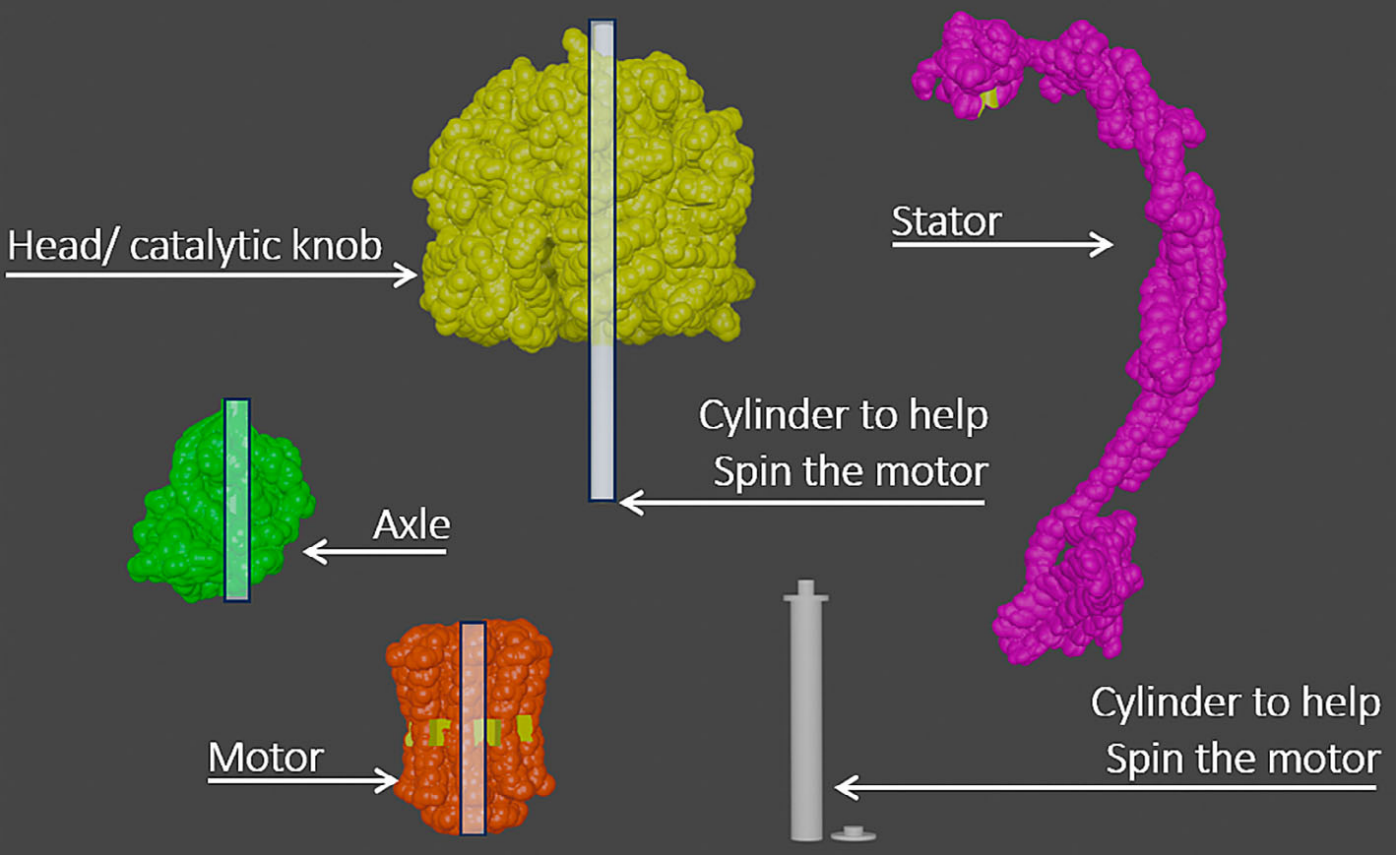
The model with simplified shapes and a mechanism to be assembled. The white parts (poles and tunnels inside the biochemical models) were created for the model to be able to move.

**FIGURE 3:**
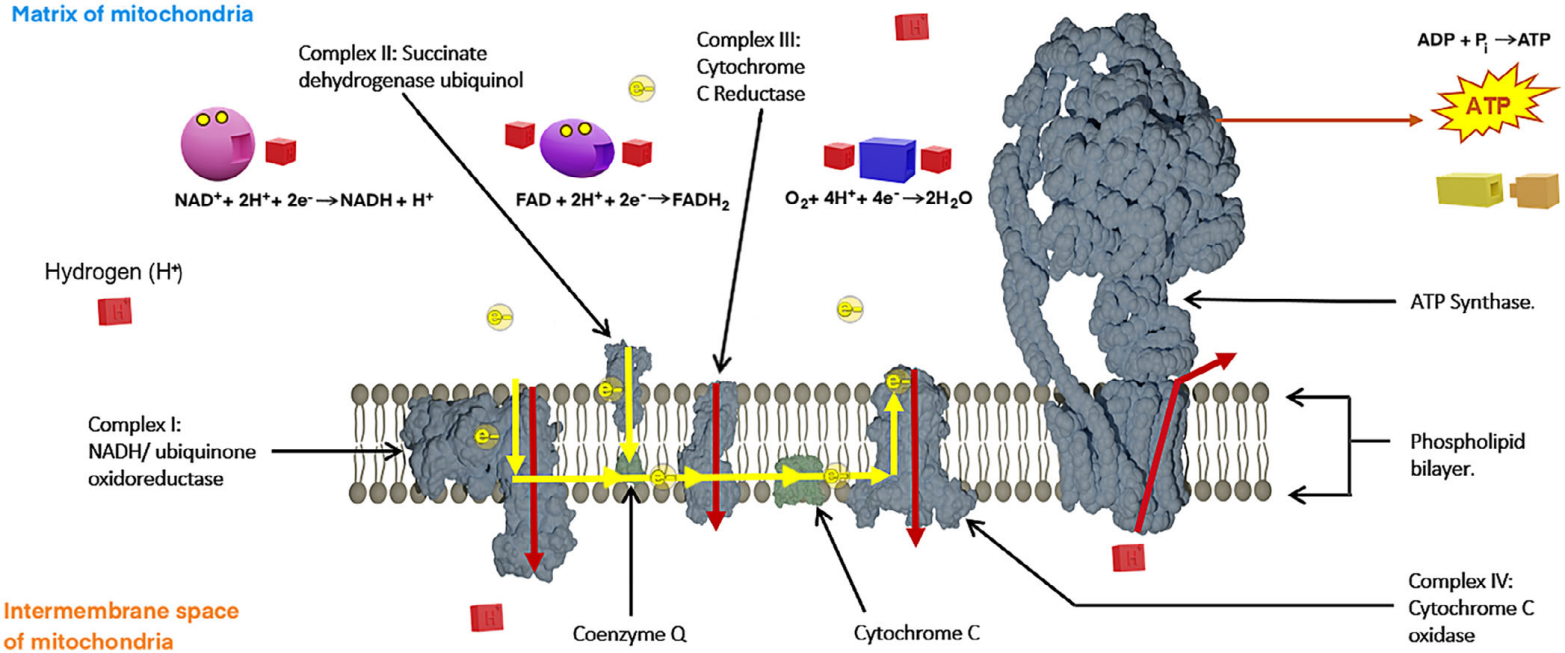
Set of chemical reactions in the electron transport chain and ATP synthesis.

In the illustrated book *Machinery of Life* (Goodsell, 2009), Goodsell paints his render of ATP Synthase in shades of red, blue, and orange, and describes it as “a molecule‐sized generator that converts electrochemical energy into chemical energy.” During chemical reaction, the large molecule combines the adenosine diphosphate (ADP) molecule with the phosphate molecule (P_*i*_) in its catalytic knob (head). Other parts of the molecular machine are made of the spinning axle and motor below it, which also provide entrances for Hydrogen molecule. These three stacked structures are stabilized by a stator connected to the catalytic knob. The individual parts of the ATP Synthase can be seen in Figure 2. The ATP creation process is preceded by an entire chain of reactions involving protein channels and cytochromes embedded in the phospholipid bilayer dividing the inside and outside of the mitochondria. Other than the electrons transferred within the phospholipid bilayer through cytochromes and protein channels, the protein channels also allow transport of Hydrogen from one side of the organelle to the other side with a different charge. On top of that, molecules such as NAD+, FAD, and Oxygen combine with Hydrogen to create NADH, FADH_2_, and H_2_O molecules that release electrons that enter the electron transport chain. For the sake of clarity and to suit the intended target audience, the installation focuses on key molecules involved in the electron transport chain and ATP synthesis, represented through physicalizations, animation, and diagrams. This choice was made deliberately to reduce complexity and emphasize the overall process by highlighting conceptual inputs and outputs rather than detailed internal pathways. Consequently, certain chemical components were omitted, such as the prosthetic groups *within* electron transport complexes that play a role in electron transfer (e.g., iron sulfur clusters, hemes, and copper centers). The entire process is represented in a simple illustration in Figure 3.

### 3.3 Previous iterations

Empirical evidence has shown that playful education has a positive effect on the learners. Examples specifically in biochemistry include tales that rely on narrative storytelling (Surapaneni, 2024) or activity‐based learning for public outreach (Roth et al., 2023). Visual literacy (Schönborn & Anderson, 2006; Schönborn & Anderson, 2010) has been proven as an essential part of understanding biochemistry and visual storytelling has been proven to help disseminate scientific findings (Botsis et al., 2020). These practices also inspired the iterations of previously published studies (Pokojná, Kriglstein, et al., 2024a) that led to the current design of this installation, discussed in detail in later (Section 3.4).

**FIGURE 4:**
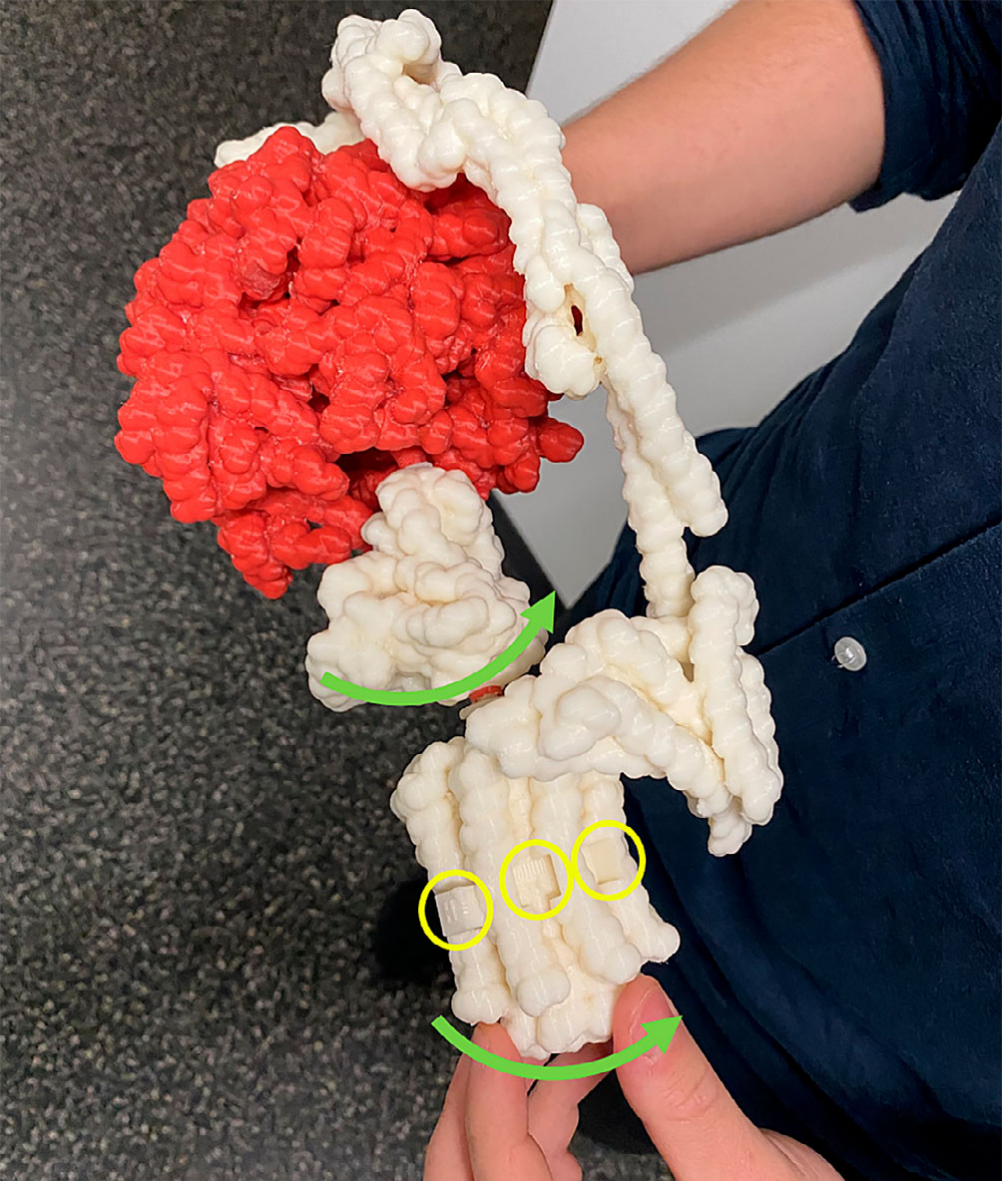
A photograph of a participant interacting with the first iteration of this design by spinning the motor of the ATP Synthase molecule (green arrow). The motor also has crevices (in yellow circles) shaped like cubes specifically for small parts that represent Hydrogen molecules. The ATP Synthase enzyme was tested as a standalone product at this initial design stage.

The first design, shown in Figure 4, consisted of a 3D physical tangible prototype of the ATP synthase, the most prominent enzyme in the ATP reaction. It was based on PDB (Berman et al., 2000) molecular data and was further adjusted in 3D modeling software (e.g., Online Community Blender, 2018) so its components can rotate as in real life (Figure 2). Its movements resemble the motions depicted in Johnson's (Johnson, 2015) or Berry's (Berry & Tétaz, 2018; Berry, 2015) molecular animations. Doing this allowed us to tackle the common issue with 3D physical models, which are accurate but rigid by default, and do not show any movement. It was then presented at a data visualization conference focusing on biology, where we collected feedback from experts in biology, medicine, and visualization, through a short survey. Overall, this representation was well received (Pokojná, Kriglstein, et al., 2024a), as it made previously inaccessible scientific phenomena both tangible and engaging. The main takeaway message from the first iteration was to create an entire transport chain, not just one enzyme concluding the series of extensive reactions.

**FIGURE 5:**
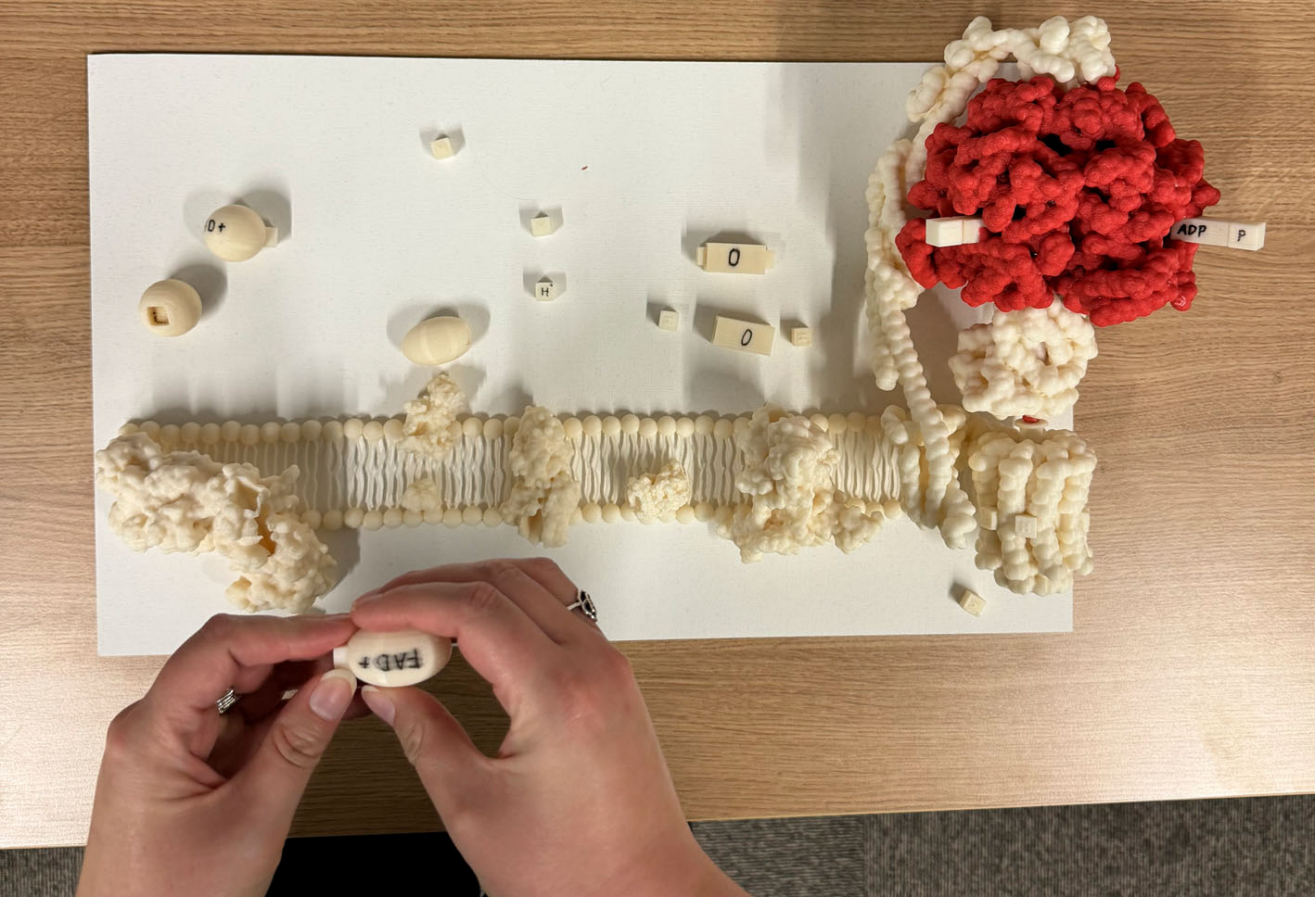
A photograph of a participant interacting with the second iteration of this design. This second design consisted not just of the ATP Synthase enzyme (right) but also the electron transport chain and molecules involved. Here, the participant puts a small Hydrogen proton in the shape of a cube into a 3D‐printed oval of an FAD molecule to create an FADH_2_ molecule.

Our second iteration, shown in Figure 5, was inspired by experts' feedback from the first ATP Synthase testing. The suggestion that we applied in our second iteration was to expand the apparatus and show the entire electron transport chain, as it is closely linked to the ATP Synthesis. The design was tested at Researchers' Night, an event where research institutions across Europe open their doors to the general public and present their research. The collected feedback (Pokojná, Kriglstein, et al., 2024a) was once again positive, and some of the suggestions included representing the electrons exchanged between the molecules. The main direction for our next iteration was to combine three modalities: illustrations, animation, and 3D physical models to reap the benefits of all three in order to show this set of biochemical processes.

Participant feedback was purposefully chosen as an indicator for the next step of the iteration because the presented artifacts were meant to evolve in the way users would see as most beneficial. Hence, formal testing with specific knowledge questions and user scales is yet to be carried out once the prototype is fully developed.

### 3.4 Installation

The *MRDAET* prototype is a collection of enhancements from the initial iterations that is scaffolded by the previous positive feedback on interaction, tangibility, and movability of the model. The details and justification for using physical objects to represent chemical reactions are discussed in previous work (Pokojná, Kriglstein, et al., 2024a). In summary, the use of 3D printed models helps with spatial understanding in the chemistry domain (Biner et al., 2018; Stieff et al., 2012). This is supported by research that found usability of physical models to be similar to virtual chemistry models (Stull et al., 2013). The additional modalities that were used in this installation, the third iteration, include illustrations and an animation overlay. Illustrations and diagrams used for biochemistry have resulted in students' better conceptual understanding compared to just using textual descriptions (Offerdahl et al., 2017; Adnan, 2020). Scientific animations affect viewers' thinking and motivation (Barak et al., 2011) and include examples from Drew Berry, whose work is intended to both teach and aesthetically appeal (Berry, 2007; Berry, 2015). Animated representations convey molecular dynamics, such as binding or protein conformations, more clearly compared to static images (Jenkinson, 2018). Molecular animations also improve information retention and motivation (McClean et al., 2005). Moreover, the use of alternative ways of visualizing text‐heavy material (e.g., animations (Taylor et al., 2007)) are also beneficial for people with reading difficulties, such as dyslexia, because they offload mental load by making information visible and touchable (Sweller, 2011). Hence, we decided to combine all three modalities into a multimedia resolution data with interactive components to obtain the benefits of all three modalities, as it makes science information more accessible in a multitude of ways.

We would like to emphasize that this type of visualization is designed to convey the science and principles of the electron transport chain and ATP synthesis to non‐experts in biochemistry, inspired by the difficulty (Dreyfus et al., 2014; Gayford, 1986; Wu & Shah, 2004; Green et al., 2021) of understanding the subject at high school level (Jones et al., 2014). As a result, certain details that would be obvious or essential for biochemists may be omitted, prioritizing clarity and helping the audience grasp the fundamental concepts without being overwhelmed.

**FIGURE 6:**
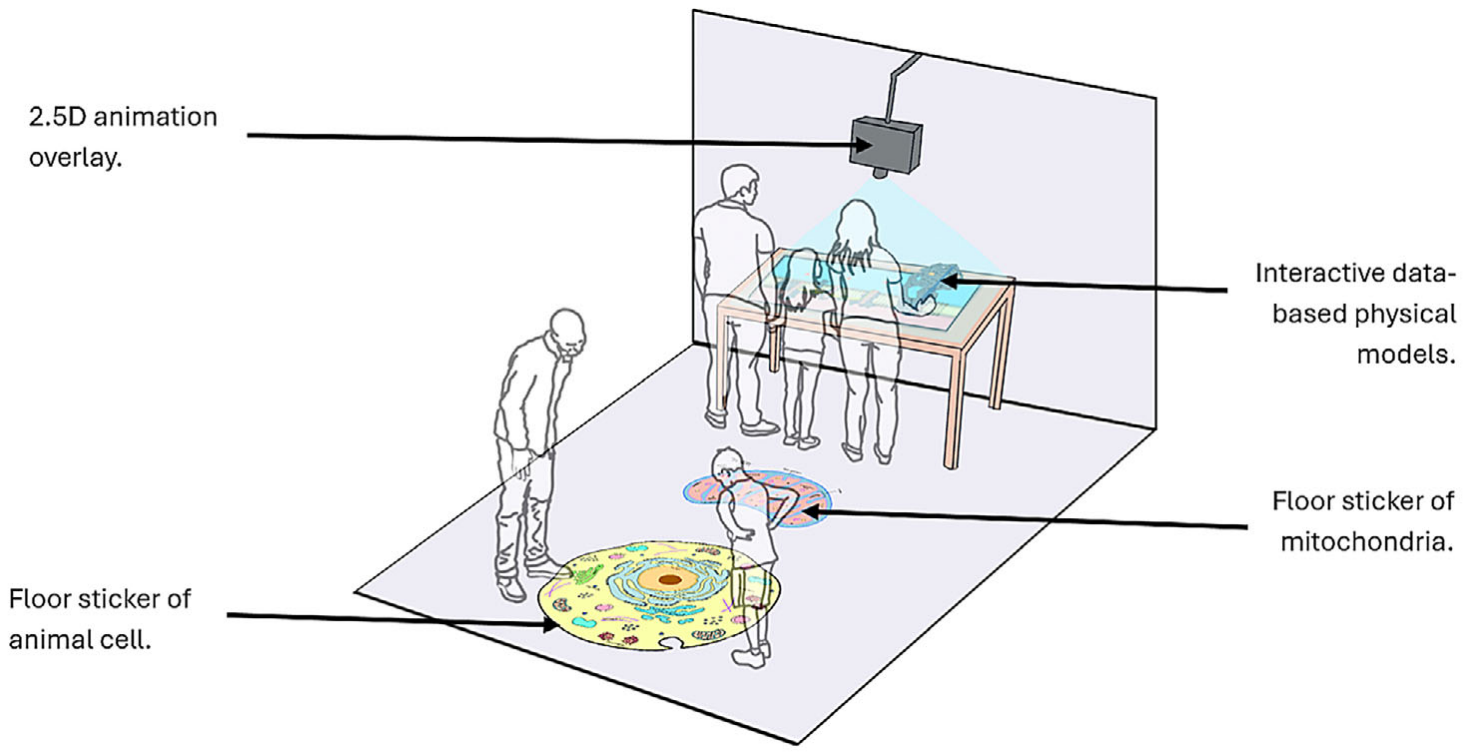
The layout of *MRDAET* installation containing large‐scale illustrations leading to 3D physical interactive models with 2.5D animation overlay. Image based on previous publication (Pokojná, Kriglstein, et al., 2024a).

In addition to utilizing the previously tested 3D physicalization (Pokojná, Kriglstein, et al., 2024a), the next step was to improve it. This consisted of adding color to the physical models, incorporating 2.5D video overlay, including magnets to represent electron transfer, and floor stickers, which contributed to the *MRDAET*'s overall unique setup of “walking into” the cell. The apparatus is deliberately designed to increase in complexity (information and visual) as the user progresses to facilitate retention (Offerdahl et al., 2017). Figure 6 shows the design of the installation.

**FIGURE 7:**
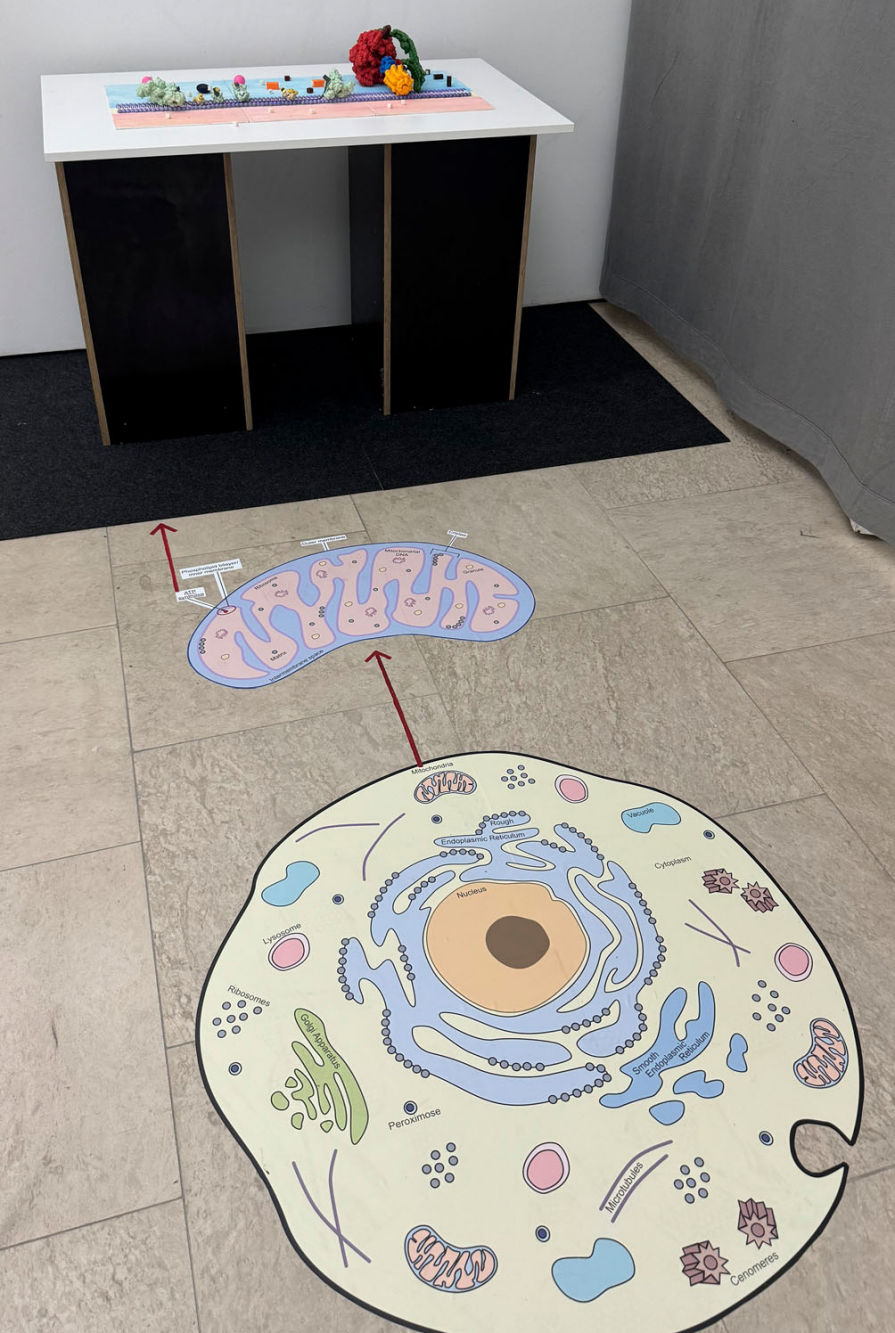
Setup of the installation consisting of a 2D illustration of the cell (lowest magnification) and mitochondria (higher magnification) on the ground. The table carries the 3D printed interactive apparatus of the electron transport chain and ATP synthesis (highest magnification), enhanced with 2.5D animation overlay.

We have created an “entrance” to the apparatus through a large, 1 m × 1 m floor sticker. The colorful, 2D illustrated diagrams with the names of components of an animal cell and mitochondria were intended to help the learners realize how small the scale of chemical reactions is and where they take place, as scales and relations of molecules and cells have been problematic to understand (Jenkinson, 2018). The overall design is set up (Figure 7) in a way where the viewer walks into the animal cell (lowest magnification) by observing a cell diagram, then continues to increase the magnification onto mitochondria by exploring another large floor sticker. The size of this installation was carefully considered as the benefit of larger physicalizations is adding an “extra” value by adding metaphorical meaning (García & Hornecker, 2022). This zooming‐in effect continued as the viewer approaches the table within the interactive components with the highest magnification, focusing on a mitochondrial wall. The design has immersive components by “walking into the cell” that also makes the display more attractive, which increases the motivation to learn (Mayer, 2014). The zooming‐in was inspired by the ARTIS‐Micropia museum's (ARTIS Zoo, 2014) beginning of entrance through an elevator that simulates increased magnification under the microscope. The installation was deliberately set up this way as the physical environment impacts people's thinking (Perovich et al., 2023).

**FIGURE 8:**
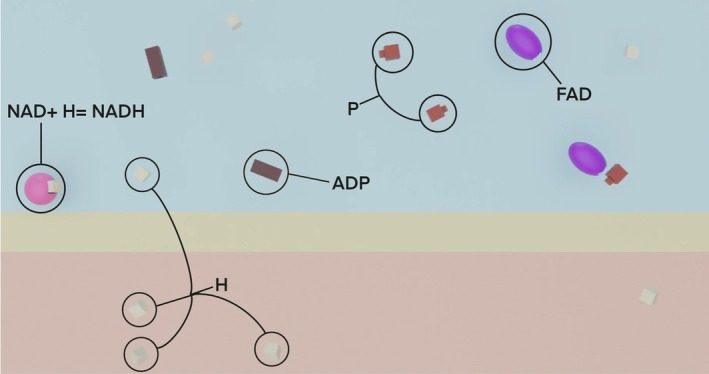
A screenshot of the 2.5D overlay video projected over the 3D physical models.

The second part of the installation is the animation overlaying the entire 3D printed apparatus. This overlay with 2.5D animation enhances the 3D physical models and is intended to illustrate how busy the two sides of the mitochondrial wall are by showing molecules moving throughout the Inter‐ and Intracellular space. Augmenting 3D molecular prints with projection was previously successfully used to depict molecular movement (Brenner, 2015; Gillet et al., 2004; Gillet et al., 2005). Figure 8 shows a screenshot of the video used in *MRDAET*: the blue background represents the Intramembrane space of mitochondria separated from the orange Intermembrane mitochondrial space by the yellow stripe—the phospholipid bilayer. The phospholipid bilayer is not visually detailed as its structure was provided by the 3D printed model that the animation was overlaying. The Inter and Intra mitochondrial spaces both have Hydrogen (white cubes), and the Intramembrane space contains pink spherical NADH, purple ellipsoid FADH_2_, red cuboid Phosphate, and brown prism ADP molecules. The models in the animation corresponded with the shape and color of the 3D physical models overlaid with this animation.

**FIGURE 9:**
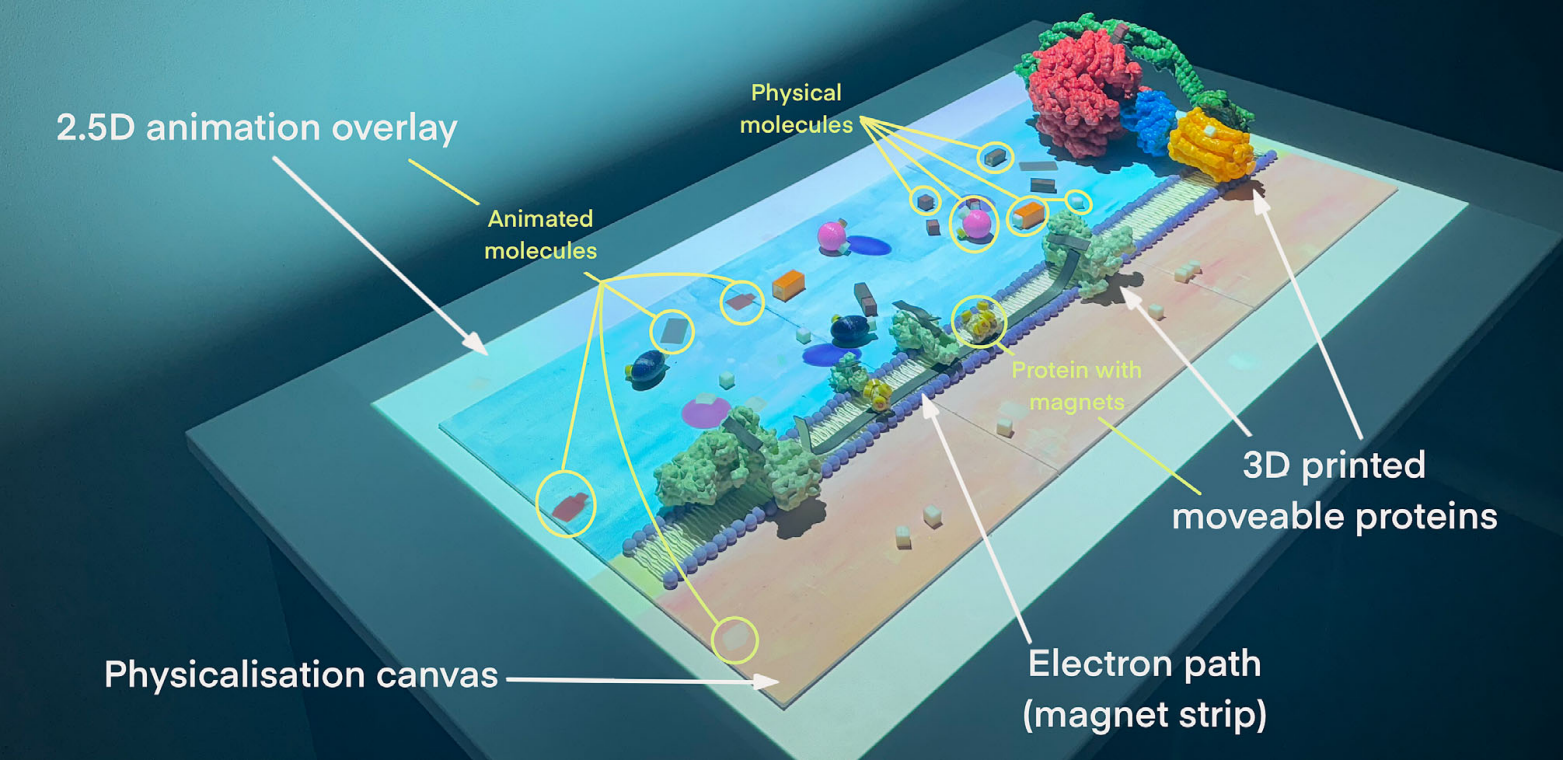
Setup of the 3D printed chemical machines included in the electron transport chain and ATP synthesis. They are placed on a board, representing the mitochondrial wall, and overlaid with animation.

The third part, made up of the 3D physical models, served as the centerpiece of this installation because of their benefits outlined in Section 2. Compared to the previous iterations, the 3D physical models shown in Figure 9 were enhanced with colors, inspired by the Goodsell coloring schemes (Goodsell, 2009), to (1) distinguish between models, (2) show the individual structures' functional similarity to each other, and (3) point to an important part of a multi‐colored model by choosing semantic colors (Schloss, 2024).

Color design for illustrative visualization (Wang et al., 2008) was previously employed in visual bioinformatics to distinguish between particles within molecules as well as to show hierarchy (Waldin et al., 2019; Waldin et al., 2016). Hues were used to distinguish between different types of structures with different functions (e.g., protein channels and H_2_O molecules). For example, the NAD+ and FAD molecules both carry electrons that were released to the electron transport chain upon binding with one Hydrogen proton. The FAD and NAD+ molecules are not only shaped similarly (round oval and sphere) but also colored with analogous colors (purple and pink). Similarly, despite there being four different protein channels in the electron transport chain, they have a similar function (to allow electrons to pass through the phospholipid bilayer) and hence they are grouped by the same hue. However, the protein channels (turquoise) and cytochromes (beige) also share a similar function to transfer electrons within the electron transport chain; hence, they were colored with less saturated colors, which visually grouped them into the electron transport chain step. This is opposed to the ATP synthase, which concludes the ATP synthesis after the electron transport chain step. This model applied visual hierarchy principles (Tufte & Graves‐Morris, 1983) by using several highly saturated colors within the same structure—red for the catalytic knob, blue for the axle, yellow for the motor, and green for the stator. These vivid colors emphasized the enzyme's importance, visually distinguishing it from its less saturated surroundings, such as neighboring proteins in the electron transport chain and the broader cellular environment, including the membrane interior, exterior, and the phospholipid bilayer. The use of four different colors also pointed out four different functions of the four different parts of the enzyme. Lastly, red and yellow semantic colors (Schloss, 2024) were purposefully used to guide the user to the catalytic knob and motor of the enzyme, where the most important chemical reactions take place, and where they should interact with the model by inserting other molecular pieces, similar to cellular building blocks (Garden, 2022). Similarly, the electron binding sites were represented by metal parts and colored yellow to attract the user's attention. We have also added magnet strips between the molecules on the electron transport chain to create a path for the round yellow magnets, representing electrons. The magnets could be removed from individual molecules and placed onto other molecules to represent electron transfer. This was an important step, as this electron transfer was not shown in the previous iterations.

## 4 USER STUDY

The following section details the participants and experimental design of the conducted user study.

### 4.1 Participants

In total, the data was collected from 35 participants. Eight participants were eliminated from the experimental analysis due to missing data (specifically, signed consent). The participants were aged 19–60 (mean average = 30.083). Of the 27 participants whose data were analyzed, 12 were female, 10 male, 1 non‐binary person, and 4 people who did not specify their gender.

### 4.2 Experimental design

The data was collected through observation and a short survey at a popular international annual event focused on art and technology. The short survey and observation were chosen because they allowed us to collect qualitative data with short‐answer questions and Likert scales from people interacting with the installation at the event. The questions focused on three areas: the enjoyment of the installation (addressing the first RQ), the helpfulness of the installation in learning (second RQ), and future improvements. Examples of questions for each theme were: “Do you like the installation of chemical reactions? Please explain why (1‐3 sentences),” “How useful do you find this in learning about chemical reactions? And why?,” and “What would you improve?”

The Likert scales gave us ordinal measures (Likert, 1932; Jamieson, 2004) that accompanied qualitative data from open‐ended responses from the short‐answer questions. The short answers were then analyzed with the qualitative content analysis (Mayring, 2015) and can be found in Section 5. This approach has been previously employed in the evaluation of similar prototypes (Pokojná, Kriglstein, et al., 2024a; Pokojná, Kozlíková, et al., 2024b), as it enables rapid feedback with low participant burden. These characteristics make it particularly well‐suited for prototype evaluation and iterative testing.

The short, open‐ended questions were employed to guide participants' attention towards specific features under investigation. As the study aimed to assess the perceived effectiveness of predefined design choices rather than to measure neutral attitudes, this approach ensured that responses directly addressed the research question. While leading questions can introduce bias, in this context, they were used deliberately to support formative evaluation. Potential bias was mitigated by pairing structured questions with open‐ended qualitative feedback, allowing participants to elaborate or challenge the evoked perspective.

The event at which we presented *MRDAET* was specifically chosen for data collection because it featured technology‐art installations, attracting people who were interested in interactive displays and exploring scientific topics in non‐standard ways. These people were a representative sample of users who would choose to visit a science museum, interact with the installations, and benefit the most from such displays. As part of our research, we wanted to observe whether this visualization would get participants without a biochemical background interested in the biochemistry topic. Since this was a large‐scale international event, we had participants from various professional backgrounds, ages, and nationalities, which provided interesting insight.

## 5 RESULTS

Here, we present quantitative scores from Likert scales and themes identified with the Qualitative Content Analyses (Mayring, 2015). This consisted of grouping topics occurring in written answers from questionnaires into themes.

### 5.1 Enjoyment of the installation

**FIGURE 10:**
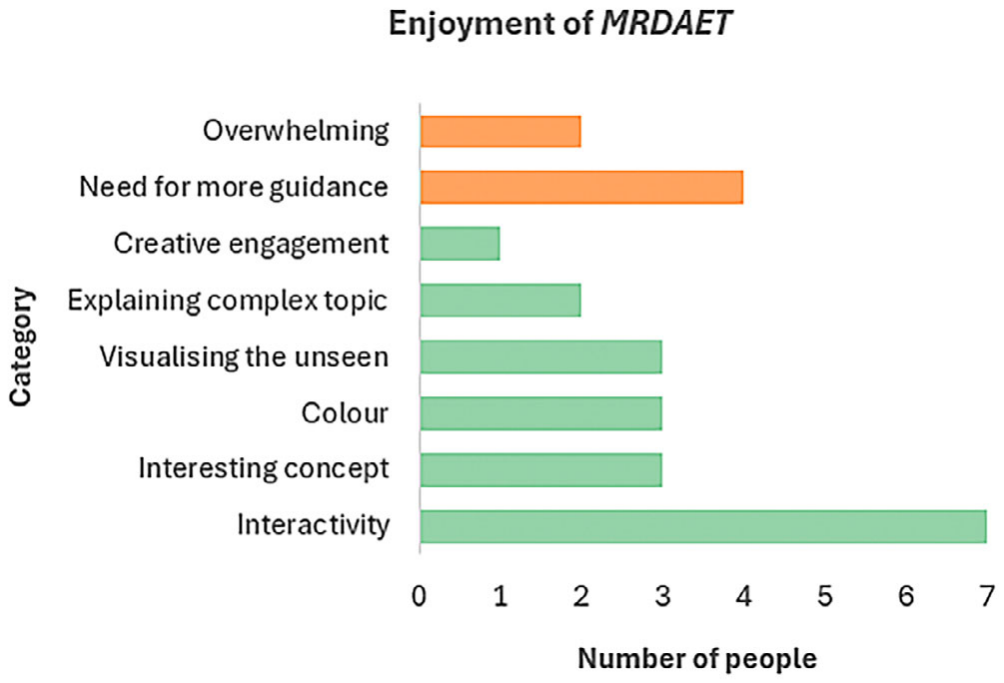
Bar chart showing identified themes for enjoyment of *MRDAET*. Green (bottom 4 bars) showing positive and orange (top 2 bars) critique categories.

The enjoyment of *MRDAET* was measured by a question: *Do you like the installation of chemical reactions? And why?* This question received an average of 4.12 out of 5 points on a Likert scale, where 1 indicated “very boring” and 5 “very fun.” The results are shown in Figure 10. The extracted themes from the short answer questions were as follows: interactivity and being able to touch the models (mentioned by 7 people), interesting concept (3 people), color (mentioned by 3 people), visualizing something otherwise unseen (3 people), making understanding of complex topic easier (2 people), and creative way to do so and engagement of the visualization (1 person). The critique included installation being too overwhelming or having too much information presented (2 people) and needing more guidance through the objects (1 person) or video explanation (3 people).

### 5.2 Helpfulness of the installation

**FIGURE 11:**
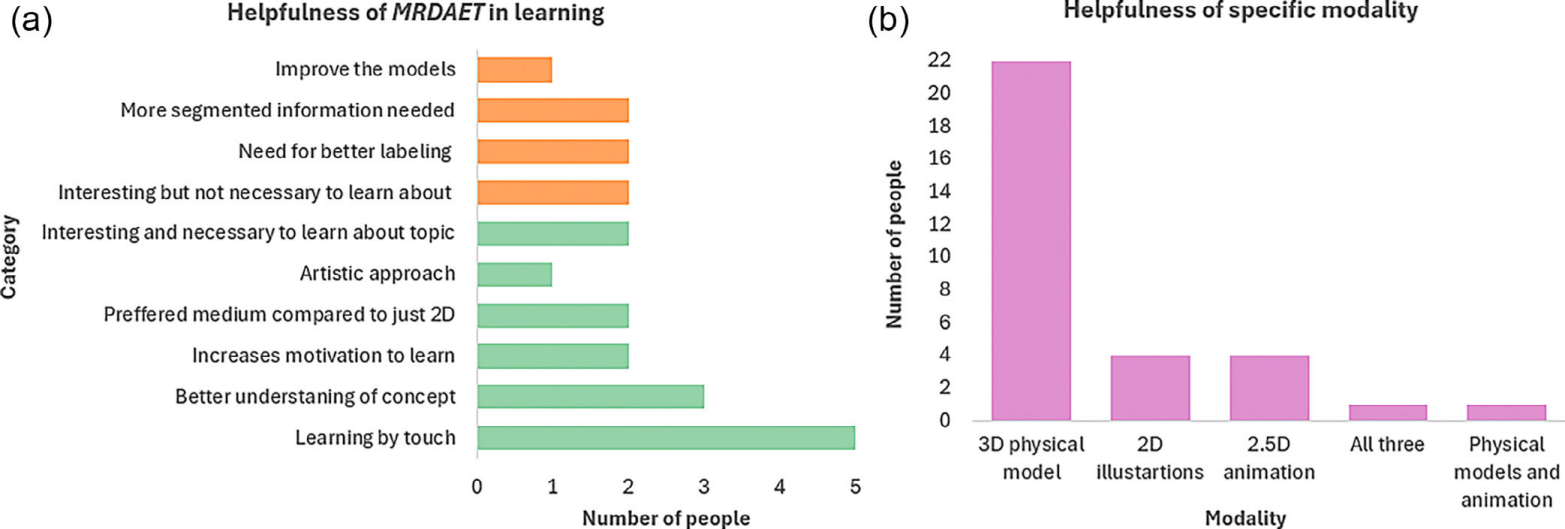
Helpfulness of *MRDAET* in (a) categories where green (bottom 6 bars) indicates positives and orange (top 4 bars) critiques; (b) shows the bar chart comparison between the usefulness of individual modalities within *MRDAET*.

The perceived helpfulness of the installation in understanding the biochemical process was measured with two short‐answer questions and multiple‐choice questions focusing on which part of the installation was most helpful and finding out whether the combined setup was better than the stand‐alone parts of the installation. In the following subsection, we summarize the outcomes for individual questions we posed to the participants. The results are also shown in Figure 11.

*How useful do you find this installation to learn about chemical reactions? And why?* The installation received an average of 4.24 out of 5 points on a Likert scale, where 1 indicated “not useful at all” and 5 “very useful.” The reasons for this were collected in the following themes: learning more by touch or playing with models (5 people), a better understanding of the concept (3 people), increased motivation (2 people), better than 2D representation (2 people), interesting to learn with the models but not necessarily about biochemistry (2 people), interesting and necessary to learn about—referring to biochemistry (2 people), and appreciation of the artistic approach (1 person). The suggested improvements comprised better labeling (2 people), further segmentation of the steps (2 people), and improvement of 3D physical models—cavities (1 person). The themes of why the installation is or is not helpful are summarized in Figure 11a.

*Which part of the installation do you find most helpful in learning about complex processes? And why?* In this multiple‐choice question, 22 people chose that the most useful component was the 3D physical interactive model, 4 appreciated a 2D illustration of mitochondria and the cell, and 4 liked a 2.5D animation of molecules. Of these, one person voted for all three, and one voted for the 3D physical models with the animation overlay. The reasoning for these choices was grouped into the following themes: touch involved in learning (9 people), 2.5D animation overlay was useful in understanding (3 people), 2D illustrations were the clearest to understand (2 people), a combination of all three modalities was most helpful to learning (2 people), spatial understanding/enzyme architecture with 3D models (2 people), enjoy going into the cell and then scaling down to mitochondrial wall (1 person).

*Do you think installation is better than stand‐alone 3D models, animation, or illustrations? Why?* Of the 25 people who answered this question, 22 people agreed that the mix of modalities is the most helpful. The reasons for this are interaction (6 people), which “brings it to life,” increased engagement (3 people), introducing fun/playful aspects (2 people), better understanding (2 people), and making it more memorable (2 people). One person mentioned that it “depends on circumstances,” and one suggested that having a teacher first walk the students through the process before showing them this installation would make it the most effective in learning. The results are also shown in Figure 11b.

### 5.3 Future improvements

The information about future improvements was collected through three questions: one short‐answer question and two multiple‐choice questions. These questions asked about what the participants would like to improve, what people find most suitable, and which type of place/institution this installation is most appropriate for.

*What would you improve?* The short answer questions collected suggestions for improvements, which were grouped into several themes. To optimize the installation experience, the participants suggested: making the animation projection as a guide, segmenting the steps more clearly, adding labels and functions to 3D physical models and models in animation (4 people), gamification, for example, adding checkpoints, making the 3D physical models more intuitive to interact with, or use of audio guide (2 people), and adding more interaction (4 people). Out of those four, two people specified interaction between animations and physical objects.

**FIGURE 12:**
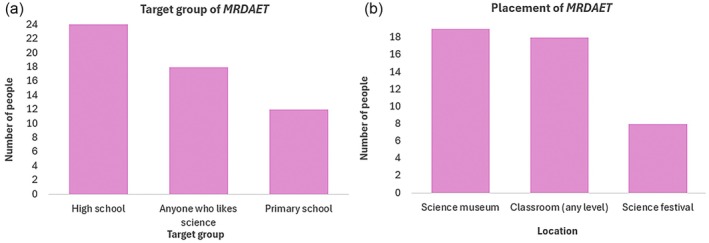
Bar charts showing aspects to be considered for real‐life application of *MRDAET*. Bar chart (a) shows the perceived target group and (b) the appropriate placement of *MRDAET*.

*What group of people would you imagine this installation for?* In this multiple‐choice question, 24 people voted for high‐school students, 18 people voted for anyone interested in science, and 12 people voted for a primary school, where two people noted that it should show simpler themes. The results are shown in Figure 12a.

*Which setting would you expect this installation to be best suited to?* In this multiple‐choice question, 19 people voted for this installation to be set in a science museum, 18 people for a classroom at the school and university level, and 8 people voted for a science festival. The results can be seen in Figure 12b.

### 5.4 Other observations

The researchers also noted down the participants' behaviors and comments. The criticism includes 4 people standing on stickers but not looking at them, 1 person asked which one of the models is protein, despite all of the physicalizations being proteins, and 15 people expected the animation to change when they interacted with the models.

The positive observations included: eight people saying that the animation is eye‐catching, one participant in particular appreciated the representation due to personal reasons because of illnesses relevant to mitochondrial malfunction.

Suggestions by participants that were said out loud and not written down included: one person suggested describing each step of a chemical reaction into a separate poster with illustration, and one suggestion to use this apparatus at Montessori School (a place where children learn by exploration from a prepared environment (Montessori, 1949)), and one person suggested making the presentation more “linear and more explicit.”

**FIGURE 13:**
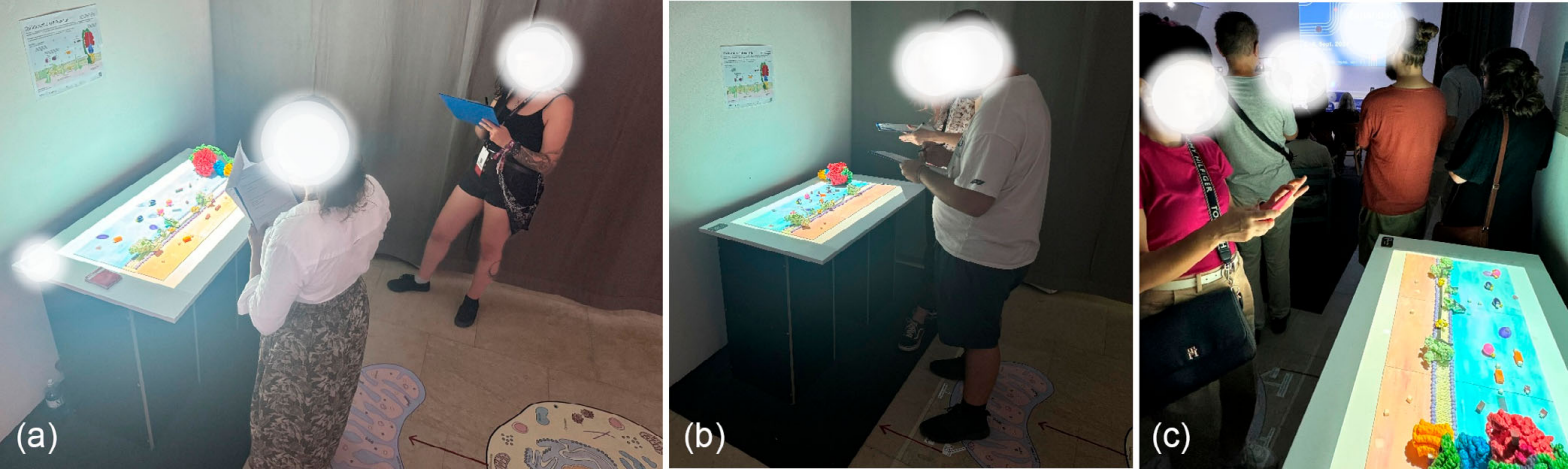
Participants observing the interactive 3D printed molecules with 2.5D animation overlay. (a) and (b) show participants filling out a paper questionnaire attached to a clipboard. (c) shows a participant taking a picture of the interactive part of the installation on the opening night.

Other observations and comments were that people who were exhibiting their works at the conference gave more feedback overall, and that the *MRDAET* reminded three people of the cartoon program for children, “Once Upon a Time…Life” (Barillé, 1987). Interestingly, five participants were observed trying to connect the models in a way they were not intended to and admitted out loud that they were looking for a way to connect the different pieces of the molecules in a way that is not described in the instructions. A photograph of participants interacting with the apparatus can be seen in Figure 13.

## 6 DISCUSSION

Here, we discuss the implications of the collected data in terms of the *MRDAET* installation regarding enjoyment, perceived helpfulness in learning, and suggested improvement.

### 6.1 RQ1: Enjoyment

The first research question was addressed with a survey question about enjoyment of the installation: *Do you like the installation of chemical reactions? And why?* Based on the responses from the participants, the enjoyment of the installation being used as a learning tool for complex biochemical processes was positive. Overall, there is an indication that the combination of these modalities successfully captured the viewers' attention and helped them understand complex and difficult‐to‐see material. Based on this feedback, we can conclude that the *MRDAET* installation provided an enjoyable learning experience. Previous studies have shown that using tangible devices for learning influences emotion positively (Li et al., 2022), as well as that the physical environment, which plays a great role in immersive experiences, has an impact on people's thought processes (Perovich et al., 2023, García & Hornecker, 2022). The enjoyment is also attributed to the aesthetics of the installation that may have removed the negative association to learning by increasing motivation through its aesthetics (Mayer, 2014). The success of interactive science museums, such as ARTIS‐Micropia (ARTIS Zoo, 2014), demonstrates the usefulness of alternative scientific education. Individual modalities, such as scientific illustrations (Offerdahl et al., 2017; Adnan, 2020), animation (Jenkinson, 2018, Iwasa, 2015), and physicalizations (Copolo & Hounshell, 1995, Alderighi et al., 2021), have been proven to be useful for information retention and motivation, by which they improve scientific education (Mayer, 2014). To the best of our knowledge, a combination of the three modalities that vary in their level of interactivity was not yet tested; hence, our contribution demonstrates that this type of visualization is worthy of further investigation.

### 6.2 RQ2: Helpfulness in learning

The second research question regarding perceived helpfulness and particular ways in which it is helpful was investigated with two open‐ended questions and a multiple‐choice question. The open‐ended questions were: *How useful do you find this in learning about chemical reactions? And why?* and *Which part of the installation do you find most helpful in learning about complex processes? And why?* The multiple‐choice question aimed to identify the most helpful component (one of the three modalities used) within the installation and asked: *Do you think installation is better than stand‐alone 3D models, animation, or illustrations? Why?* For the first question, the installation received high scores for helpfulness in learning. Most participants stated that the 3D physical models were very helpful, which shows that tactile exploration plays a role in the learning process of abstract structures (Norooz et al., 2015). This is consistent with the principles of STEAM, Exploranation (Ynnerman et al., 2018) and embodied experience (Reiner, 1999). Our results adhere to previous research, particularly the comments from participants, where 22 out of 25 participants agreed that using a combination of modalities was better than using just a single modality. This is consistent with previous findings about using multiple modalities (Krajcik & Sutherland, 2010) and learning through dual encoding (Paivio, 1986) without overwhelming the cognitive load (Sweller, 2011). The different modalities contribute to visual literacy, which is fundamental for understanding biochemical concepts through external representations such as diagrams, animations, and dynamic models (Schönborn & Anderson, 2010), and visual literacy should be taught and leveraged not just for lay people but also for biochemists (Schönborn & Anderson, 2006). Despite the physicalizations being the most preferred modality individually if one had to be chosen (22 people) relating it to learning by touch (9 people), the other modalities were also well perceived. Animation was chosen as the most helpful by four people, as well as four people voting for the illustration. Animations were helpful because they helped understand the understanding of the concept (Iwasa, 2015; Jenkinson & McGill, 2012) and illustrations were helpful as they were easy to understand (2 people), which is consistent with previous findings (Offerdahl et al., 2017, Adnan, 2020).

A lot of the learning aspect is also attributed to the interaction through touch that helped explain the architecture of proteins, their movement, and molecular interactions by making abstract concepts visible (Norooz et al., 2015). These results are also consistent with the idea of “embodied experience,” which emphasizes that learning is enhanced when people physically interact with abstract concepts, allowing them to connect mental representations with tangible actions (Reiner, 1999). This was demonstrated numerous times in scientific education (Minshall, 2019), for example, in bio‐design (Okerlund et al., 2016) or gene editing (Verish et al., 2018). Its success can be attributed to hands‐on interaction (Hutmacher & Kuhbandner, 2018; Novak & Schwan, 2021) because it enhances memory and understanding of objects. A meta‐analysis (Li et al., 2022) shows that tangible education is useful since it can influence emotions and relies on multiple senses. The same study also showed that tangible educational resources are particularly useful in collaborative settings, such as this one, as they facilitate cooperation and communication (Shaer & Hornecker, 2010; Laal & Ghodsi, 2012). However, the haptic input may be effective for learning only at the immediate time of learning and may not persist over time (Novak & Schwan, 2021). From these answers, we can conclude that people enjoy and find it useful to employ tactile learning, especially when it comes to learning about hard‐to‐visualize topics, such as molecules (Norooz et al., 2015; Schönborn & Anderson, 2010).

Other collected data pointed out that the visualization approach with multiple modalities was most helpful in learning about ATP synthesis and the electron transport chain. We attribute this to the increased engagement and playful aspects not just through tactile exploration (Norooz et al., 2015; Novak & Schwan, 2021), but also by employing attractive visuals (Mayer, 2014). Studies support that multi‐sensory experiences increase learning, whether it be in a digital environment like Virtual Reality (VR) (Philippe et al., 2020) or even the environment created for STEM education specifically for children (Tsoukala, 2021).

In practice, *MRDAET* is intended for the general public and hence only the fundamental information, such as main actors within the chemical reactions, is presented, as described in Sections 3.2 and 3.4. While maintaining accuracy is important, this type of visualization heavily relies on aesthetics and interaction in order to make the information attractive and hence evoke motivation to learn (Mayer, 2014). The goal is to lead the users to understand the basics before continuing to more difficult concepts and perhaps returning to more traditional and more detailed means of learning.

### 6.3 Lessons learned

Based on our observations, we offer several suggestions for creating similar installations in other contexts, such as different subjects (e.g., physics) or additional biochemical topics (e.g., the Krebs cycle (Kornberg, 2000)). First, we highly recommend several smaller iterations, especially for the interactive parts. Our original design consisted just of the movable ATP model, and by adding more components to it throughout several rounds of iteration, we went from a hand‐held apparatus to an interactive installation. This approach allows us to create a user‐centered apparatus tailored to their needs. This relates to our second recommendation, that suggest taking advantage of people's knowledge that can lead to improvements. Therefore, we would recommend that the design be tested at an event that combines science, art, and technology, as it attracts people who are experts in all these fields and can provide valuable feedback, where expert suggestions and user needs are discussed. At the same time, it also attracts the general public, who can aid with user design improvements from observation. Third, we recommend 3D printing with sturdy materials, such as Acrylonitrile Butadiene Styrene (ABS) instead of Polylactic Acid (PLA). We observed three participants aggressively interacting with our apparatus when the researchers were further away from the installation. At one point the molecules were assembled incorrectly, forming an indistinct, blob‐like structure, despite the diagram clearly illustrating the proper arrangement. This was reinforced by five participants who attempted to arrange the 3D models in ways they were not meant to be put together, while outwardly stating their intention.

An open‐ended question “*What would you improve?*,” combined with observational data of participants interacting with the apparatus and multiple‐choice responses (indicating target audience and site) informed the identification of areas for future development in the next iteration. In general, the themes for improvement varied, but the most prominent one was adding an interaction between the 2.5D animation and the 3D models, for example, through embedded sensors (Vincke et al., 2020), which would take it from the current augmented overlay to mixed reality (MR). MR would allow users to manipulate virtual objects anchored in the real world with a higher level of interaction (Speicher et al., 2019). Taking this direction would be the next step, as it was not only suggested by the participants in their short answers, but especially indicated by their behavior. The researcher has observed 15 participants looking for a change in animation when they interacted with the physical 3D models, similar to 3D prints changing their augmentation as in the work by Gillet et al. (2004). The benefits of this approach would be higher engagement (Guo et al., 2017), immediate feedback (Geller et al., 2021), and offering a collaboration opportunity in virtual space (Goagoses et al., 2025; Brůža et al., 2025). However, implementing this can raise several other issues. For example, having multiple people interacting in the same digital space may lead to conflicting interactions (Billinghurst et al., 2002) where one participant may carry out an action that would be undone by another participant. This issue could be solved by preventing simultaneous interactions on the same objects through turn‐taking or role allocation. Visual cues would also be helpful to indicate if an action has already been performed on a specific object. Too much engagement can also lead to less learning (Makransky et al., 2019), as the participants would prioritize immersion that would essentially be a distraction, instead of a learning tool. Another problem may arise when too many inputs would lead to participants' cognitive overload (Sweller, 2011). Several participants have already indicated that they would prefer more guidance (4 people) or that there was too much information provided already (2 people), and experienced cognitive overload (Sweller, 2011). These could be solved by segmenting the steps in the chemical reaction more explicitly (Perovich et al., 2023), for example, through posters for individual steps, also suggested by one participant. Alternative ways of segmenting the already existing information could be by adding a guided tour through video projections or an “audio tour.” These aspects would have to be considered in the early stages of planning the next, more complex iteration of *MRDAET* to be executed effectively.

Given the success of various visual media aiding in the understanding of biochemistry knowledge, we assumed that an interactive art installation grounded in real data could serve as an effective alternative for learning about biochemical processes. As the results from our prototype testing indicate, using a multimedia tool, such as this one, carries the benefits of all included modalities. These observations are consistent with previous research. The floor stickers illustrating the cell and mitochondria helped to give an overview of the structure and placement of individual components, which is in line with previous findings about diagrams employing scaffolding in order to support learning (Offerdahl et al., 2017), for example, through gradually increasing complexity and focusing on the key components. The progressive increase in magnification also supported understanding of the relative scales of the cell, mitochondria, and molecules, as such size relationships can be challenging to understand (Jenkinson, 2018). Moreover, the animation overlay helped with visualizing molecular crowding and dynamics within the cell (Jenkinson, 2018; Jenkinson & McGill, 2012) as these can be challenging to grasp. The results also indicated that the physicalizations helped with explaining the spatial placement of individual molecules in the electron transport chain, as well as the shape of individual molecules (Ferk et al., 2003). Overall, the physicalizations supported the understanding of abstract unseen concepts (Norooz et al., 2015).

### 6.4 Limitations and future work

Despite generally positive participant feedback, the prototype exhibits several limitations. A design issue that could easily be improved would be by signposting the floor stickers, as some participants were observed to simply walk over them without noticing them. An effective and visually appealing technique would be using light to emphasize the mitochondria within the cell to highlight it as a region of interest, as well as mark the beginning of the installation. The themes for improvement varied, but the most prominent one was adding an interaction between the 2.5D animation and the 3D models, which would take it from the current augmented overlay to interactive MR. This interaction could be achieved by having the 3D physical objects acting as controllers that would signal to the sound system to make a sound (Bae et al., 2023) or play a different animation (Jansen & Dragicevic, 2013). Another way to build on this project would be through interaction and gamification by adding a point system that counts every time a chemical reaction is “achieved” by connecting proper physical molecules in the right order. A meta‐analysis of science education through gamification has shown that well‐designed gamified environments can enhance motivation and engagement if correctly integrated into the learning process (Baah et al., 2024). An example of doing so would be balancing the gamification in a way that does not distract from actual learning (Makransky et al., 2019).

It is important to recognize the limitations inherent to the modalities. For example, the 3D printed models are rigid and show simple movements such as rotation and molecular reaction sites. However, a movement where the reaction site moves and the molecule's transformation and transition contribute to a follow‐up process is not easy to show in rigid 3D printed models. A specific example is the movement of Hydrogen binding to the motor of the ATP synthase and moving *from* one side of the phospholipid bilayer *to another*. This movement then helps to rotate the axle of the ATP synthase, which in turn causes the ADP and P to combine in the reaction sites in the head of the protein to create the ATP molecule. While the Hydrogen enters the motor of synthase in this iteration of *MRDAET*, the crossing of bilayer could be visualized more explicitly in a future iteration. That could involve a mechanism to move the Hydrogen and perhaps a signpost of its impact onto the head of the protein animation overlay light or some other movement of the protein head, such as quivering.

Considering the positive response from the users towards their enjoyment of the apparatus and their perceived helpfulness of it, we believe that it is worth investigating in the future in the context of science visualization. Science museum (voted by 19 people) would benefit from this the most, followed by use in classroom (18 people) and science festivals (8 people). In either case, it would benefit novices to learn about biochemistry, whether it be high‐school students (24 people), anyone interested in science (18 people), or primary school children (12 people), for example, even in Montessori school focused on learning through exploration (Montessori, 1949). The principles and combination of modalities, intentionally showing various scales, as well as using various levels of interaction, could be applied to other STEM subjects, such as biology or physics. It would be interesting to show, for example, traversing scales from matter through the atom to the nucleus or the Krebs cycle (Kornberg, 2000).

In terms of different modalities, *MRDAET* relies on interactivity and immersivity thanks to technology, but without being totally digitally immersed, like in Virtual Reality. There are many VR applications that benefit understanding concepts in biology and chemistry, such as virtual labs (Barrow et al., 2024), a combination of VR with feedback devices (Gebhardt et al., 2022), allowing a virtual tour inside of molecules (Johnson et al., 2015; Alharbi et al., 2023), or building nanostructures (Kut'ák et al., 2021). However, these applications rely on a headset to be immersed in virtual space, which is different from apparatus like *MRDAET*, which relies on immersivity through tactile exploration and visual inspection in the real world (e.g., illustrations and physical objects). While VR is a modality that is developing rapidly and is a powerful immersive visualization tool, it comes with several setbacks such as discomfort (motion sickness), limited tactile feedback, accessibility, and cost, as well as cognitive overload (Wang, 2025). VR offers valuable advantages for education and visualizations, but its limitations highlight the importance of exploring other modalities, for instance physicalizations, because they are more cost‐effective and do not require users to have advanced technical skills.

We also highlight the importance of collaboration with the domain experts (in this case, biochemistry) to prevent factual mistakes. In our installation, the physical and animated representation of the FAD molecule has a site for accepting one electron, despite the fact that the molecule accepts up to two electrons (the correct representation is depicted in Figure 3). While this inaccuracy does not significantly affect the perception of visualization and its impact on learning, it concerns objective data representation and should be addressed in future iterations. Thanks to the digital nature of these modalities, the issue can be readily corrected within the 3D modeling software before 3D printing.

## 7 CONCLUSION

In this paper, we presented *MRDAET*, a multimedia artistic tool aimed at science education. Based on our survey, we evaluated that using multi‐modal interactive approaches, such as *MRDAET*, in educational settings (e.g., science museums) would benefit the users who are new to the hidden world of biochemistry. We posed questions to explore the perception of the installation that shows science through art. To answer RQ1 concerning enjoyment, the combination of several modalities makes it enjoyable to interact and retain information. This would be especially helpful for novices in the topic, whether it be the general public or high school students who struggle with grasping these concepts. Regarding RQ2, which focused on perceived helpfulness in learning, the interaction with 3D physical models, enhanced through a 2.5D animation overlay and supported by clear, intuitive visual cues, made this multimodal approach particularly valuable for education. It addressed several key challenges in molecular learning: understanding complex spatial arrangements, visualizing the movement of otherwise unseen molecules, representing the dynamic and crowded nature of the cell, clarifying the spatial location of processes within it, and the grouping of molecules by function.

The input from participants about the improvement of the tool helped us determine the next iteration of the installation., The participants' feedback has guided us through three iterations. The unexpected, yet most helpful comments were regarding the 2.5D animation by adding interactivity through the 3D models and music, or a guide to the experience. Another component that could be added would be gamification, which would increase mental stimulation and incentive to learn. The findings further suggested overarching guidance for such interactions, such as providing more direction to the participant and preparing the interactive pieces for heavy use by using sturdy materials. We believe that multimedia interactive installations represent a valuable addition to STEAM education by offering an engaging alternative for learning about abstract scientific processes. Based on the collected user feedback, our findings suggest that multimodal visualizations combining art and science have the potential to enhance learning through interactivity and aesthetic appeal and warrant further development and a more detailed study.

## AUTHOR CONTRIBUTIONS

**Hana Pokojná:** Conceptualization; investigation; writing – original draft; methodology; validation; visualization; writing – review and editing; formal analysis; project administration; data curation; funding acquisition; resources. **Barbora Kozlíková:** Writing – review and editing; supervision; writing – original draft. **Katarína Furmanová:** Writing – review and editing; supervision; writing – original draft. **Adam Štěpánek:** Writing – review and editing. **Simone Kriglstein:** Conceptualization; writing – review and editing; supervision; writing – original draft.

## Data Availability

Research data are not shared.
